# Extranodal Lymphoplasmacytic Lymphoma Responding Rapidly to Zanubrutinib: A Case Series

**DOI:** 10.1002/ccr3.71756

**Published:** 2026-01-02

**Authors:** Samuel Brown, Jillian Rusbridge, George Follows, Anna Santarsieri

**Affiliations:** ^1^ Clinical School of Medicine University of Cambridge Cambridge UK; ^2^ Department of Haematology Addenbrooke's Hospital Cambridge UK; ^3^ Cambridge University Hospitals NHS Foundation Trust Cambridge UK

**Keywords:** chronic diseases, hematology, oncology, pharmacology and pharmacy

## Abstract

Management of recurrent or atypical lymphoplasmacytic lymphoma poses a significant therapeutic challenge. Here we demonstrate a case series of three patients with atypical presentations of extranodal lymphoplasmacytic lymphoma that each show remarkable and timely responses to Zanubrutinib with a limited side effect profile.

## 1 Introduction

Lymphoplasmacytic lymphomas (LPL) are a rare, incurable group of non‐Hodgkin lymphoma (0.27 per 100,000 people in the USA [1]). They arise from post‐germinal centre B‐cell lymphoproliferation with, usually, IgM monoclonal gammopathy. When IgM paraprotein is produced, this disease can be termed Waldenström's Macroglobulinemia (WM) [2]. The median survival is 5.3 years for individuals > 75 years [3]. More than 95% of WM demonstrate an L265P mutation in the adaptor protein MYD88 (Myeloid Differentiation Primary Response 88) involved in IRAK1 and NF‐κB signaling, promoting cell survival [4]. This mutation leads to the constitutive homodimerisation of MYD88 and subsequent recruitment and activation of several intracellular molecules, including Bruton Tyrosine Kinase (BTK) [5].

The symptoms of LPL are largely due to bone marrow infiltration and associated cytopaenias, commonly with constitutional symptoms such as weight loss and fatigue [2], as well as moderate hepatosplenomegaly, lymphadenopathy, and anemia. Occasionally, platelet dysfunction or coagulopathy may be observed due to paraproteinaemia [2]. The IgM paraprotein can also lead to blood hyperviscosity, which increases the risk of headaches and strokes and may induce an inflammatory response and cause neuropathies [6].

Management of symptomatic patients with LPL generally includes Rituximab as monotherapy, or in combination with chemotherapy [7]. These therapies confer good 2‐year progression‐free survival (PFS) of 89% with Rituximab and Bendamustine and 81% with Bortezomib, Dexamethasone, Rituximab, and Cyclophosphamide in a recent meta‐analysis [8]; but chemotherapy is associated with side effects including gastrointestinal symptoms, cytopenias, risk of infections, and neutropenic sepsis, and the longer‐term risk of secondary cancers [9]. Also, there is a limited number of lines of chemotherapy that can be used due to cumulative toxicity.

More recently, BTK inhibitors (Ibrutinib, Zanubrutinib) have been licensed for LPL and Zanubrutinib has been approved by NICE for treatment of patients with relapsed LPL [10]. BTK inhibitors reduce the pro‐survival downstream signaling from dysregulated MYD88 (L265P) homodimers [4]. These targeted therapies have revolutionized LPL treatment, with a high response rate and favorable safety profile [11].

Here we present three patients with rare extranodal manifestations of WM, each with the MYD88 L265P mutation, and assess their response to the BTK inhibitor Zanubrutinib.

## 2 Case History and Examination

### 2.1 Patient A

Patient A was first diagnosed with WM in 2011 at the age of 76 years, having presented with night sweats, weight loss, generalized debility, lymphadenopathy, and bone marrow failure. He had an IgM paraprotein of 17 g/L, and bone marrow trephine biopsy revealed a lymphoplasmacytoid infiltrate of > 50%. He underwent four cycles of fludarabine‐cyclophosphamide combination chemotherapy, which he completed in January 2012. He subsequently relapsed and completed four cycles of Rituximab‐Bendamustine chemotherapy in September 2014. Treatment was stopped at this point due to pancytopenia, and he subsequently suffered from recurrent infections. He was commenced on immunoglobulin replacement therapy for panhypogammaglobulinaemia. He remained in remission from lymphoma until 2022, when he had an atypical, rare relapse.

### 2.2 Patient B

Patient B was diagnosed with WM in December 2021, at the age of 69 years. She presented with exertional breathlessness and pancytopenia. She initially required a blood transfusion, and bone marrow trephine biopsy demonstrated 60% infiltration with LPL. MYD88 L265P mutation was detected by PCR. She had a low‐level IgM paraprotein of 11 g/L. A staging CT scan revealed no lymphadenopathy or splenomegaly. Treatment with Rituximab monotherapy was commenced, and she completed four cycles. She had an excellent response to treatment with normalization of the hemoglobin, a fall in the paraprotein to < 2 g/L. However, in November 2022, the WM began to relapse with pancytopenia and a rise in the IgM paraprotein to 7 g/L. A restaging CT scan revealed multiple enlarged abdominal and left pelvic lymph nodes. She was treated again with four cycles of Rituximab monotherapy and prednisolone, and once again responded well to treatment, until 2023 when, like patient A, she presented with a rare, but distinct relapse.

### 2.3 Patient C

Patient C, unlike patients A and B, had a rare, atypical presentation of WM as his first presentation, rather than as a relapse. He was diagnosed with WM in May 2023 at the age of 70 years, having presented with a gradual decline in mobility and cognition over 12–18 months.

## 3 Investigations and Treatment

### 3.1 Patient A

**FIGURE 1:**
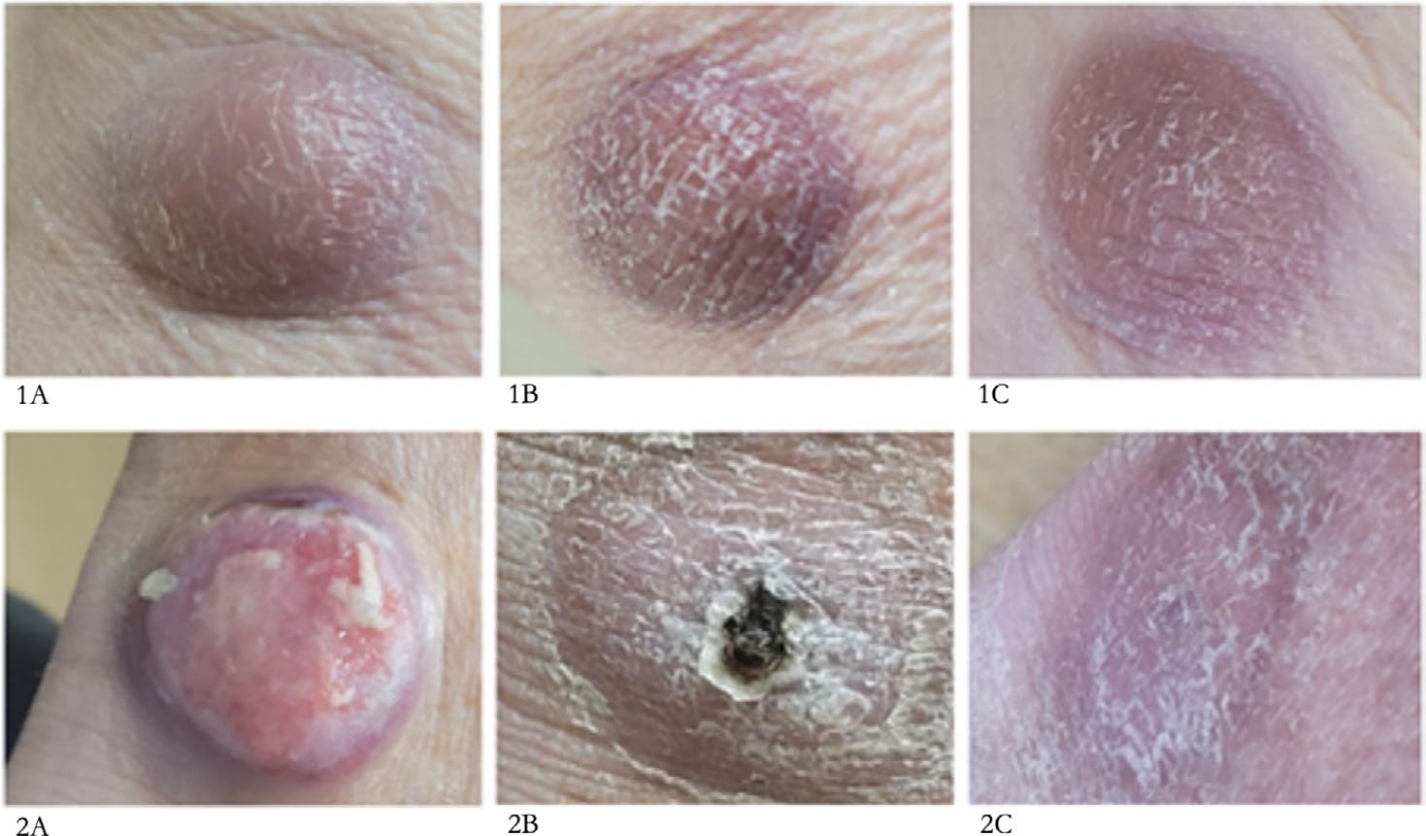
Cutaneous involvement with lymphoplasmacytic lymphoma in Patient A, pre‐ and post‐Zanubrutinib treatment. (1) lesion from posterior left knee (2) lesion from left ankle taken: (A) pre‐Zanubrutinib, (B) 4 weeks post start of Zanubrutinib and (C) 8 weeks post start of Zanubrutinib.

In 2022, patient A developed an ulcer on his left ankle with associated cellulitis, which, when biopsied, was in keeping with a low‐grade lymphoma. On analysis, it was confirmed that the MYD88 L265P mutation was present, as with his original lymphoma; however, there was no detectable paraprotein in his blood. He underwent a CT scan, which revealed multiple cutaneous and subcutaneous lesions on both sides of the diaphragm. Given widespread disease, radiotherapy was not a treatment option. As Ibrutinib had stopped being funded on the NHS for LPL, the decision was made to wait for Zanubrutinib to become available. Throughout the year, he developed further skin lesions (Figure 1) that came and went, predominantly on his lower legs, and small, palpable groin nodes, though his blood counts remained stable, still with no detectable paraprotein. When reviewed again in August of 2023, the clinical picture was the same, with no palpable lymphadenopathy or hepatosplenomegaly. His blood counts were stable, but he still had firm nodular lesions, and the non‐tender, indurated erythematous purple plaques that they would leave behind. These plaques were now also noted on his abdomen, left chest, and left upper arm. Biopsy of a plaque on the left medial thigh in October 2023 confirmed that they were, as suspected, the LPL, without transformation.

When reviewed in clinic in January 2024, it was evident that the nodules on his lower legs were interfering with his daily activities. In particular, a recent lesion on his left medial ankle was open and weeping, having rubbed on footwear. He also exhibited an itchy rash on his chest, with pink macules of approximately 1 cm. The decision was made to start treatment with Zanubrutinib, 320 mg daily, along with an antihistamine.

### 3.2 Patient B

**FIGURE 2:**
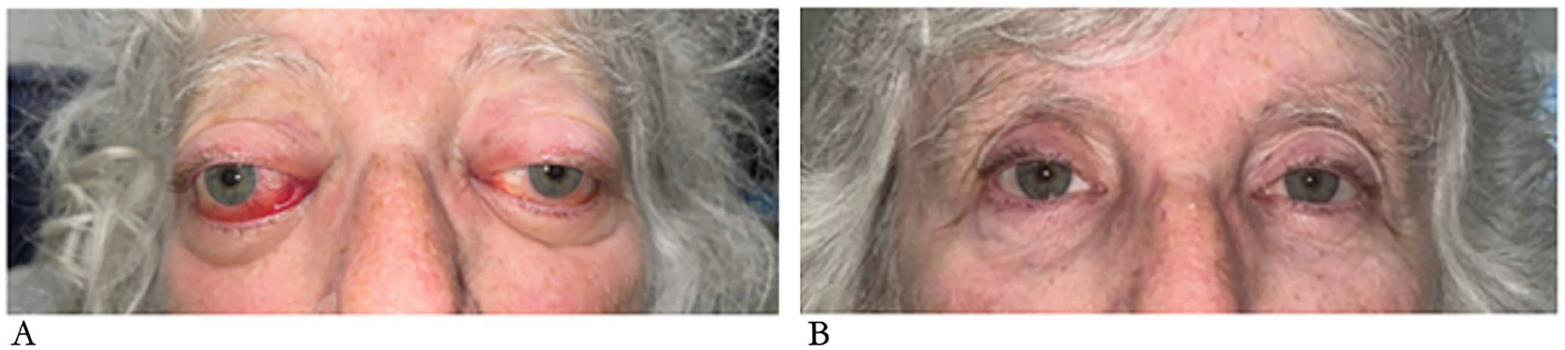
Orbital infiltration with lymphoplasmacytic lymphoma in Patient B, pre‐ and post‐Zanubrutinib treatment. (A) pre‐Zanubrutinib, (B) 12 weeks post start of Zanubrutinib.

In October 2023, patient B presented with bilateral proptosis (Figure 2) and binocular vertical diplopia. Examination in the ophthalmology clinic revealed right superior oblique weakness with secondary left inferior rectus under action. There was also bilateral restriction of abduction and elevation with a restriction of right adduction. An urgent MRI of the brain and orbits demonstrated soft tissue infiltration bilaterally in the superior intra‐orbital regions with no evidence of brain involvement. Right orbital biopsy was performed in December 2023 and confirmed recurrent LPL with no evidence of transformation. At this time, her hemoglobin was normal and < 2 g/L IgM paraprotein was detectable. A restaging CT scan showed small‐volume enlarged lymph nodes in the right lower cervical and axillary regions and a mesenteric mass which had reduced in size compared with her previous imaging and measured 45 × 17 mm. Zanubrutinib was commenced in December 2023, at a dose of 320 mg daily.

### 3.3 Patient C

MRI head revealed unusual nodular leptomeningeal infiltration and an extra‐parenchymal paranasal ethmoid mass. The diagnosis of LPL was confirmed on bone marrow examination and on biopsy of the ethmoid mass, with presence of MYD88 L265P mutation. He had a low‐level IgM paraprotein of 4 g/L. Although Patient C did not undergo a brain biopsy and no lymphoma cells were found in the cerebrospinal fluid (CSF), it was felt highly likely that he had Bing Neel syndrome (BNS), with leptomeningeal involvement with LPL. The proximity of the biopsy‐proven LPL to the central nervous system (CNS) in the ethmoid further supported the diagnosis of BNS. BNS is a rare presentation, caused by CNS involvement by LPL [12]. BNS is usually diagnosed by LPL findings on biopsy of cerebrum or meninges, which Patient C declined, or LPL cells in CSF [12]. A baseline PET‐CT scan identified scattered metabolically active lesions within the axial skeleton, but no lymphadenopathy or hepatosplenomegaly.

There is no standardized treatment for BNS. Treatment options were discussed including high‐dose methotrexate and intrathecal methotrexate versus Zanubrutinib, which can be accessed in specific circumstances for the first line setting for LPL using private healthcare insurance. Although responses have been reported in patients treated with immunochemotherapy and with intensive high‐dose methotrexate‐containing protocols used in high‐grade CNS lymphoma, toxicity is high and response rates are modest [13]. There have also been reports of successful treatment of BNS using Zanubrutinib [14] and as this drug penetrates the CNS it has been investigated in clinical trials in primary CNS lymphoma. He chose to start treatment with Zanubrutinib.

## 4 Outcome and Follow‐Up

### 4.1 Patient A

Patient A observed a reduction in the size of the cutaneous lesions after day 4 of Zanubrutinib, and the ulcerating lesion on his ankle had begun to heal. By day 12, when reviewed in clinic, the patient estimated that the skin lesions had halved in size and reported no side effects. When reviewed again 5 days later, however, he reported lethargy (grade 2) and anorexia (grade 1), so was advised to halve the dose of Zanubrutinib to 160 mg daily. His pruritis resolved, and on day 26 of treatment, when reviewed again in clinic, he continued to exhibit a profound reduction in size of the lesions (Figure 1).

After 8 weeks of treatment the cutaneous lesions had continued to regress, leaving behind only skin discoloration. The patient chose to increase the dose back to 160 mg twice daily and felt lethargic once again. In cycle 3 he was advised to reduce the dose to 160 mg daily; however, in the fourth cycle the skin lesions increased in size, and he increased the dose back to 160 mg twice daily, which he continued for cycles 5–10. The patient then developed a chest infection and had a treatment break for 6 weeks. He restarted Zanubrutinib at a dose of 80 mg daily and has continued with this dose for 9 months to date. He is now 18 months into treatment with Zanubrutinib and is tolerating the treatment well, albeit at a reduced dose. The skin lesions have healed and not recurred; his blood counts are stable and there is no detectable paraprotein.

### 4.2 Patient B

When seen for a 2‐week follow‐up, patient B's eyes were less erythematous and swollen, but the diplopia remained. She had experienced no side effects of Zanubrutinib and was feeling well. At 4 weeks the diplopia was improved, and she was prescribed a further two cycles of Zanubrutinib 320 mg once daily. At 3 months after starting Zanubrutinib, sequential exophthalmometry demonstrated an improvement in the proptosis from 30 mm and 27 mm (pre‐treatment) to 18 mm and 17 mm (after 3 months of Zanubrutinib); the diplopia had resolved, and her vision was 6/6 in both eyes. The patient is now 20 months into treatment with Zanubrutinib. She continues taking the standard dose and tolerates it well. There has been no recurrence of the proptosis and the paraprotein is undetectable.

### 4.3 Patient C

**FIGURE 3:**
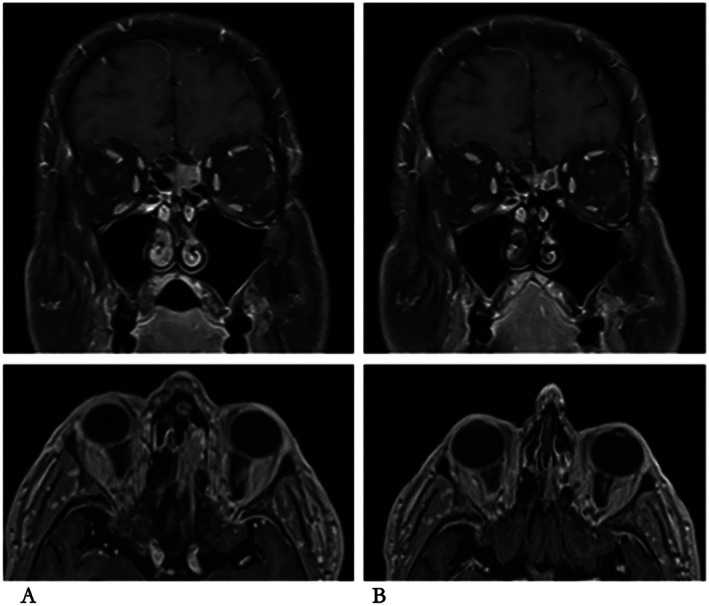
Ethmoidal and leptomeningeal involvement with lymphoplasmacytic lymphoma in Patient C. MRI head pre‐ and post‐Zanubrutinib treatment. (A) pre‐Zanubrutinib in coronal section (above), and axial section (below). (B) 3 months post start of Zanubrutinib in coronal section (above), and axial section (below).

Within the first month, Patient C experienced an improvement in his memory and in his mobility and balance. After 3 months of Zanubrutinib 320 mg daily, he had an MRI head which showed that both the leptomeningeal disease and ethmoid mass were responding (Figure 3). The interim PET‐CT scan showed reduction in fluorodeoxyglucose uptake at all skeletal sites in keeping with a complete metabolic remission. He continues treatment with Zanubrutinib and has an ongoing excellent response at 30 months and is in a complete remission on MRI. He has tolerated the treatment well and has a very mild sensory neuropathy affecting his feet but reports no other side effects.

## 5 Discussion

We present three cases of extranodal manifestations of LPL that have shown remarkable response to Zanubrutinib. Our key findings are two‐fold. Firstly, we document unusual cases of both relapses and first presentation of this malignancy, which we hope will enable consideration of LPL as a differential for future patients. Secondly, we demonstrate effective treatment of these patients with minimal side effects.

In all three cases there was very low or undetectable paraprotein in the presence of clinically significant extranodal disease. These cases are rare and there is limited literature on the dissociation between paraprotein and disease burden which we have observed. A retrospective study of patients with LPL and non‐IgM paraprotein or no paraprotein showed a higher prevalence of extramedullary disease and a lower rate of MYD88 mutations than in WM. However, patient survival outcomes were similar [15].

There has been good evidence for Zanubritinib as a selective BTK inhibitor in the treatment of typical LPL [16], and a growing amount of literature for atypical presentations of LPL in extranodal sites [17, 18, 19]. There is however limited evidence for the use of Zanubrutinib in extranodal LPL, which we have demonstrated with these cases as an effective and relatively safe option to be considered. We present Patient A as a case of cutaneous WM trialed on Zanubrutinib, showing excellent response. Cutaneous WM encompasses IgM bullous disease, IgM‐storage papules, and neoplastic cell infiltrates [17]. There are few reported cases of cutaneous presentations of LPL, and so limited literature on the use of Zanubrutinib to treat cutaneous presentations. This patient observed a rapid reduction in tumor size from the first few days of treatment. They reported fatigue: a well‐documented side effect of Zanubrutinib [20], which was managed successfully with dose reduction.

By comparison, in 2020, a retrospective study [21] characterized the disease in nineteen patients with WM with cutaneous manifestations. Amongst those without transformation, skin lesions preceded diagnosis in two cases, in four cases the lesions were concomitant with diagnosis, and six cases with WM preceded occurrence with a median delay of 3.5 years. Non‐transformed WM patients developed plaques (83%), nodules or tumors (42%), or papules (25%). A systematic review has been published looking at primary macroglobulinemia‐induced immunobullous dermatosis [22], however, this article also included ‘IgM monoclonal gammopathy of undetermined significance’ as well as WM patients. One case report of cutaneous presentation demonstrated a good response to Ibrutinib, sustained at 18 months [23], but with severe toxicities that led to the choice of the more selective, next generation Zanubrutinib in its place [16].

Patient B presented with a history of WM, diplopia and proptosis. MRI revealed intraorbital soft tissue infiltration with effect on extra‐orbital muscle action, and she was trialed on Zanubrutinib. There are very few reports of an orbital infiltration presentation in the literature; those reported are usually bilateral. Treatment for orbital infiltration is poorly defined, but has previously included solely chemotherapy, or a chemoradiotherapy combination [18]. Patient B showed marked improvement within the first month of starting Zanubrutinib, and at 3 months her diplopia had resolved. A systematic review [18] detailed 18 cases of different ocular manifestations, including unilateral and bilateral orbital masses, infiltration of rectus muscle and lacrimal gland involvement. Another case [24] details a patient with orbital mass and extra‐ocular muscle involvement, who went on to be treated with ibrutinib monotherapy, followed by rituximab, bortezomib and dexamethasone following disease relapse. This patient remained stable for over 3 years before orbital swelling recurred.

Orbital involvement in LPL has also been demonstrated in patients with BNS [25, 26], a rare manifestation involving infiltration of LPL of the CNS causing neurological deficits [12]. One case report described treatment with Ibrutinib leading to resolution of bilateral optic nerve swelling. Other cases of orbital involvement of WM in association with BNS differed in their treatment regimens (without the use of Zanubrutinib), with some success [25, 26, 27]. This contrasts with other cases in which, despite aggressive chemotherapeutic treatment, patients had only partial resolution or suffered severe vision loss or total blindness [28, 29].

While patient B did not display evidence of having BNS, Patient C did have a clinical picture and investigations consistent with BNS: namely, a decline in mobility and cognition combined with nodular leptomeningeal infiltration. Bone marrow biopsy confirmed MYD88 L265P mutation and histology consistent with LPL. The marked improvement in neurological and MRI signs following Zanubrutinib in this patient provides further evidence for this treatment for BNS. Elsewhere in the literature, in a case series of 44 WM patients, BNS developed at a median time of 4 years from diagnosis, although in 36% of these patients BNS was the first manifestation [19] of WM. Ibrutinib has been shown in a cohort study of 28 patients to lead to an 85% improvement or resolution of BNS symptoms and 47% clearance of disease from CSF [30].

There has also been a report of Zanubrutinib as a monotherapy [14] following unsuccessful treatment of WM in a 75‐year‐old female with rituximab, cyclophosphamide, vincristine, and prednisolone. She re‐presented with difficulty walking, and subsequent investigation revealed contrast‐enhancing lesions on MRI and CSF showing an increased total protein and lambda light chain restricted monoclonal B cells. She was diagnosed with BNS and received 12 cycles of high‐dose intravenous methotrexate. Unfortunately, the neurological dysfunction worsened, and she was trialed on 160 mg BD Zanubrutinib, which led to marked neurological improvement and maintained IgM paraprotein below 2 g/L at 15 months.

To summarize these comparisons, there are very few previous accounts of Zanubrutinib being used for management of these manifestations of this malignancy. In the rare reports of these cases, there are reports of the use of ibrutinib to varying effect [23, 24, 30]. The findings of this series are therefore promising as an alternative treatment, and we recommend further research to be conducted into the use of Zanubrutinib.

## 6 Conclusion

We present here three cases of a rare malignancy, lymphoplasmacytic lymphoma, with extranodal manifestations that are rarer still, namely: cutaneous, optic and leptomeningeal. Not only do we provide case studies of reference for these unusual presentations but also provide insight into the ability of the next‐generation BTK inhibitor, Zanubrutinib, to rapidly and successfully treat them. Two of these patients were treated in relapse, one as first line, but all exhibited rapid and complete improvement (though will continue to be monitored long‐term). Moreover, in contrast to chemotherapy, side effects were minimal if any, including fatigue and mild peripheral neuropathy. These results are encouraging, and we recommend that further studies with larger patient cohorts should be conducted on the therapeutic benefit of Zanubrutinib, particularly in relapsed, atypical cases of LPL.

## Author Contributions

**Samuel Brown:** writing – original draft. **Jillian Rusbridge:** writing – original draft. **George Follows:** writing – review and editing. **Anna Santarsieri:** writing – review and editing.

## Funding

The authors have nothing to report.

## Consent

Written informed consent was obtained from Patients A, B, and C to publish this report in accordance with the journal's patient consent policy.

## Conflicts of Interest

The authors declare no conflicts of interest.

## Data Availability

Data sharing not applicable to this article as no datasets were generated or analyzed during the current study.
